# Role of lipid composition on the structural and mechanical features of axonal membranes: a molecular simulation study

**DOI:** 10.1038/s41598-019-44318-9

**Published:** 2019-05-29

**Authors:** Marzieh Saeedimasine, Annaclaudia Montanino, Svein Kleiven, Alessandra Villa

**Affiliations:** 1 Department of Biosciences and Nutrition, Karolinska Institutet, Huddinge, Sweden; 2 Division of Neuronic Engineering, KTH-Royal Institute of Technology, Huddinge, Sweden

**Keywords:** Computational biophysics, Membrane structure and assembly

## Abstract

The integrity of cellular membranes is critical for the functionality of axons. Failure of the axonal membranes (plasma membrane and/or myelin sheath) can be the origin of neurological diseases. The two membranes differ in the content of sphingomyelin and galactosylceramide lipids. We investigate the relation between lipid content and bilayer structural-mechanical properties, to better understand the dependency of membrane properties on lipid composition. A sphingomyelin/phospholipid/cholesterol bilayer is used to mimic a plasma membrane and a galactosylceramide/phospholipid/cholesterol bilayer to mimic a myelin sheath. Molecular dynamics simulations are performed at atomistic and coarse-grained levels to characterize the bilayers at equilibrium and under deformation. For comparison, simulations of phospholipid and phospholipid/cholesterol bilayers are also performed. The results clearly show that the bilayer biomechanical and structural features depend on the lipid composition, independent of the molecular models. Both galactosylceramide or sphingomyelin lipids increase the order of aliphatic tails and resistance to water penetration. Having 30% galactosylceramide increases the bilayers stiffness. Galactosylceramide lipids pack together *via* sugar-sugar interactions and hydrogen-bond phosphocholine with a correlated increase of bilayer thickness. Our findings provide a molecular insight on role of lipid content in natural membranes.

## Introduction

Axons are long projections of the nerve cell that are characterized by an excitable plasma membrane. In myelinated axons, patches of axon membrane are wrapped into myelin sheath, which enables a more efficient transmission of electrical signal^1^. The exposure to excessive stress can cause damage of the cellular membrane or myelin sheath, resulting in axon’s dysfunctions that can be the origin of neurological diseases^2–4^. Knowing how these cellular elements respond to deformation is necessary to better understand their role in the axon.

**Figure 1:**
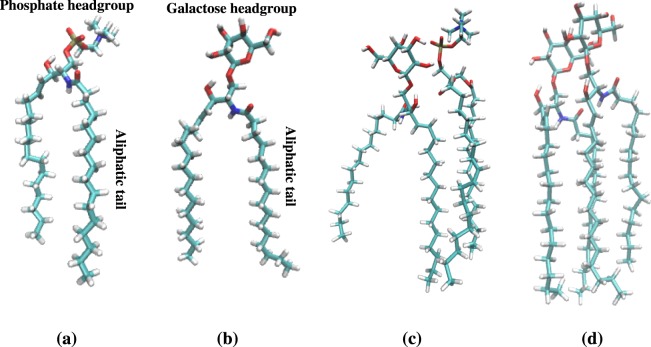
Lipid structures and interactions: (**a**) sphingomyelin and (**b**) galactosylceramide lipid molecules. Examples of lipid-lipid interactions (**c**) between galactosylceramide and phosphatidylcholine lipids and (**d**) between galactosylceramide lipids in *GalCer-rich* membrane. Carbon atoms are colored in cyan, oxygen in red, hydrogen in white, nitrogen in blue, and phosphorus in brown.

Electron microscopy studies have shown that the myelin sheath is composed of a multilayered stack of thick membranes with electron-dense and electron-light layers wrapped around the axon^5^. The dry mass composition of myelin is characterized by a high proportion of hydrophobic lipid molecules (70–85%) and a low proportion of protein (15–30% hydrophobic proteolipid protein)^6^. Myelin lipid composition is special and differs from the composition of other types of membranes (Table 1). The most striking feature is the enrichment of myelin with a cerebroside-type of lipid, beta-D-galactosylceramide (galactosylceramide or GalCer), that is characterized by having *β*-D-galactose as headgroup (Fig. 1b). The concentration of cerebrosides in the white matter is directly proportional to the amount of myelin content (around 28%)^7^. In addition to cerebroside, the other main components are cholesterol (CHOL) and phospholipids.

**Table 1:** Lipid composition of the simulated bilayers and natural membranes in weight fraction.

	POPC	POPE	CHOL	SM	GalCer	POPS	Others
**Bilayer Models**							
*Pure-POPC*	1 (1)	0	0	0	0	0	0
*POPC*/*POPE*	0.51 (0.50)	0.49 (0.50)	0	0	0	0	0
*Reference*	0.42 (0.35)	0.40 (0.35)	0.18 (0.3)	0	0	0	0
*SM-rich*	0.24 (0.2)	0.22 (0.2)	0.18 (0.3)	0.36 (0.3)	0	0	0
*GalCer-rich*	0.24 (0.2)	0.23 (0.2)	0.19 (0.3)	0	0.34 (0.3)	0	0
**Natural Membrane**							
*Red blood cell* ^7^	0.17	0.18	0.23	0.18	0.03	0.07	0.14
*Myelin* ^7^	0.10	0.15	0.22	0.08	0.28	0.09	0.08

The corresponding mol fractions are reported in parentheses. For abbreviations see (a).

(a): POPC = 1-palmitoyl-2-oleoyl-sn-glycero-3-phosphocholine; POPE = 1-palmitoyl-2-oleoyl-sn-glycero-3-phosphoethanolamine; CHOL = cholesterol; SM = sphingosine-phosphorylcholine; GalCer = beta-D-galactosylceramide; POPS = 1-palmitoyl-2-oleoyl-sn-glycero-3-phospho-L-serine.

Although considerable information is available on the electrical properties of the myelin, less is known on its structural features and on the role of the lipid compositions^8^. Membrane lipid content usually plays significant role in membrane structural feature and adhesion^9^. Experimentally, the membrane structural properties can be investigated using X-ray scattering^10^ and nuclear magnetic resonance (NMR) spectroscopy^11^, while its mechanical properties can be determined using micropipette aspiration on lipid vesicles^12^ and atomic force microscopy (AFM) on supported lipid bilayers^13^ or pore-spanning membranes^14^. Micropipette aspiration experiments conducted on lipid vesicles showed that the lipid degree of saturation and the cholesterol concentration mostly affect the membrane stiffness^15–17^. AFM experiments showed that cholesterol and sphingolipids enhance the mechanical resistance of lipid bilayers^18^. These techniques have micron scale resolution and limitations: optical imaging limits micropipette aspiration, tip size and temperature dependence limit AFM. This results in large variation of reported values for mechanical stiffness of membranes and cells^19^ and makes systematic comparison of lipid bilayers very difficult.

Alternatively, molecular dynamics (MD) simulations can be used to investigate the effect of lipid content on membrane properties in a systematic way and at molecular resolution. The benefit of MD simulations is that the contribution of each lipid type to the structural and mechanical properties can be individuated^20^. Molecular simulations have been widely used to elucidate how cholesterol and lipid types influence membrane structure and dynamics^21–23^. Both atomistic (AA) and coarse grained (CG) simulations have been used to clarify the response of membrane to the mechanical stress: *i.e*. by applying mechanical tension to the membrane^24–26^ or by applying an unsteady deformation to the lipid bilayer^27^. Simulations have been also used to rupture the membrane. For example, Tieleman *et al*.^28^ and Leontiadou *et al*.^25^ reported that the 90 mN/m stress is enough to form an irreversible breakdown in a phospholipid bilayer; Groot *et al*.^29^ determined the rupture properties of mixed bilayer using dissipative particle dynamics and showed that by increasing the mole fraction of the surfactant, the lipid bilayer could withstand smaller surface tension prior to the rupture. Shigematsu *et al*.^27^ studied the mechanical rupture of phospholipid bilayers containing different concentrations of cholesterol and showed that the critical strain for pore formation increases when the cholesterol concentration is 40%.

Here, we aim to elucidate the role of lipid content on the structural and mechanical properties of a membrane. We use MD simulation techniques and two leaflets of lipids (lipid bilayer) to describe the membrane. To mimic myelin-lipid content, we use a lipid bilayer containing 30% GalCer, 30% cholesterol molecules, and the rest phospholipids (labelled as *GalCer-rich* in Table 1), while plasma membrane content is described by a lipid bilayer containing 30% N-palmitoylsphingosine-phosphorylcholine (sphingomyelin or SM) in place of GalCer (labelled as *SM-rich* in Table 1). As phospholipids, we use 1-palmitoyl-2-oleoyl-sn-glycerol-3-phosphocholine (POPC) and 1-palmitoyl-2-oleoyl-sn-glycero-3-phosphoethanolamine (POPE), since they are the dominant phosphocholine and phosphoethanolamine lipids in plasma membranes and myelin sheaths (Table 1) ^7^. For comparison, a POPC/POPE/CHOL bilayer, labelled as *Reference* (Table 1), and bilayers, containing only phospholipids, have also been studied. Cholesterol content has been shown to influence the membrane structure and stiffness^11,16^, thus we keep it constant to 30% in all CHOL-containing bilayers to avoid extra effect on the results.

Using bilayer models that differ only for one lipid type allows us to distinguish the effect of a specific lipid type on the bilayer properties and ultimately helps us to understand the role of each lipid to the mechanical response (stress) of the natural membranes. Note that the simulated lipid bilayers are simplified models of cellular membranes: they do not account for the proteins embedded in the cellular membranes, for the variety of lipid types and for the asymmetry of the cellular membranes.

To the best of our knowledge, there are no mechanical simulation and/or experimental data available neither on selected membrane composition, nor on the myelin membrane. Lack of experiment on myelin sheath might be due to the difficulty in obtaining an *in vitro* myelin model that could be experimentally manipulated^30^.

In the following, we report the simulation results for the bilayer models. We have simulated the bilayers using an atomistic (CHARMM)^31^ and coarse-grained (MARTINI)^32,33^ descriptions and using different system dimensions (having 7 × 7 nm^2^ and 42 × 42 nm^2^ as bilayer area) to avoid bias due to model description and size. To extract membrane mechanical properties, we have performed simulations at constant surface tensions. First, we report the structural features at equilibrium and compare with the available experimental data. Then we discuss the mechanical properties. To allow comparison with experimental data, mechanical properties have also been evaluated for cholesterol-less phospholipid bilayers. At last, we look at how the structural feature and water permeability are affected by mechanical stress. All the results together support that having 30% of GalCer in the lipid content increases membrane area compressibility modulus.

## Methods

### System setup

The membranes were modelled as a lipid bilayer, where both leaflets have the same lipid composition. Different lipid compositions were used (see Table 1 for composition details). The so-called *Reference* bilayer is composed of 70% phospholipids and 30% cholesterol molecules. *SM-rich* and *GalCer-rich* membrane models have the same amount of cholesterol (30%) but less phospholipids than the *Reference*, instead they have 30% SM and GalCer, respectively. Table 1 reports the lipid mole fraction for different membrane models. The fractions have been chosen based on the major lipid compositions of red blood cell plasma membrane and myelin membrane^6,7^. The atomistic structure of the lipid and cholesterol molecules are shown in Fig. S1. *Pure-POPC* and *POPC/POPE* bilayers (Table 1) were used as a matter of comparison with experimental data and to mimic the lack of cholesterol molecules, when needed.

The initial coordinates of atomistic and coarse-grained bilayers were constructed using the membrane-only generation option of the Membrane Builder in CHARMM-GUI^34^. A symmetric lipid distribution was applied. A initial box of 7 × 7 × 12 nm was used. Each bilayer contains around 200 lipid and cholesterol molecules. The bilayers were hydrated with 4 nm-thick water layer to avoid the effects of periodic images in the normal direction to the bilayer. An ion concentration of 150 mM NaCl was used. A box of 42 × 42 × 12 nm coarse-grained membrane was build from the equilibrated structure of the CG (7 nm) system. Each CG (42 nm) system contains around 7200 lipid and cholesterol molecules.

The membrane systems were simulated at the atomistic and coarse-grained levels. The CHARMM36^31^ force field together with TIP3P water model^35^ were used for atomistic simulations, while the MARTINI2.2^32,33^ force field together with one-bead non-polar water model^36^ were used for CG simulations. In the MARTINI model, small groups of atoms (3–4 heavy atoms) are united into beads which interact with each other by means of empirical potentials. No CG models corresponding to the atomistic SM and GalCer were available, instead the corresponding “general” models were used: the CG SM model has C(d 18:1/16:0) hydrophobic tail (with 2 carbon atoms less than atomistic model) and the CG GalCer model has C(d 18:1/16:0) hydrophobic tail (with 2 carbon atoms more than atomistic model) (see Fig. S1 for details). In the following the membrane models will be labelled according to the molecular model (AA for atomistic and CG for coarse grained model), composition, and box dimension in x/y directions (Table S1).

### Molecular dynamics simulations

All MD simulations were performed using the GROMACS simulation package^37^, version 2016 (manual.gromacs.org/#latest-releases). The bilayer systems were equilibrated at constant temperature (37 °C) and pressure (1 bar). The pressure was held using semi-isotropic Parrinello-Rahman barostat^38^ with a time constant of 5 ps (compressibility of 4.5 × 10^−5^ bar^−1^) for AA and 12 ps (compressibility of 3 × 10^−4^ bar^−1^) for CG simulations. The Verlet cutoff scheme was used^39^. The AA simulations were run up to 0.6 *μ*s using 2 fs timestep. All bonds containing hydrogen atoms were constrained using the LINCS algorithm^40^. The electrostatic and van der Waals interactions were calculated using Particle Mesh Ewald method^41^ with a real-space cutoff of 1.2 nm. The temperature was maintained using the Nose-Hoover thermostat^42^ with a coupling constant of 1 ps. The CG simulations were run up to 50 *μ*s (for 7 nm bilayers) and 3 *μ*s (for 42 nm bilayers) using 20 fs timestep. Electrostatic forces were calculated using a reaction field potential^43^ with cutoff at 1.1 nm in line with MARTINI setting. Van der Waals interactions were calculated between all beads separated by less than 1.1 nm using cutoff potential. The temperature of the systems was maintained using velocity rescale thermostat^44^ with a time constant of 1 ps.

To evaluate membrane behavior under mechanical stress we performed simulations at constant surface tensions (NP_*z*_*γ*T ensemble). In these simulations, we coupled the pressure in x and y directions to a constant surface tension in the plane of the bilayer and the pressure in the z direction was coupled to 1 bar using Berendsen pressure coupling^45^. For the surface tension, values of 0, 2, 5, 10, 20, 30, 40, 50, 60, and 70 mN/m were considered, except for *Pure-POPC* and *POPC/POPE* (see Table S1 for details). To monitor the pore formation, we have also simulated CG lipid bilayers (42 nm) at constant areal strain (NP_*z*_AT ensemble) for different strain values.

### Simulation analysis

To characterize a lipid bilayer, we calculated area per lipid, bilayer thickness, carbon order parameters, cholesterol orientation, hydrogen bonds, number of lipid contacts, solvent accessible surface (SAS)^46^, lipid interdigitation and water permeability. Reported values were averaged on the last 0.2 *μ*s for atomistic simulation and on the last 10 *μ*s and 1 *μ*s for small and large CG simulations, respectively. The errors were obtained by dividing the data production into four parts and calculating the standard error between them. Carbon order parameters, cholesterol orientation, hydrogen bonds, and SAS have been calculated only for AA simulation data.

The average area per lipid was calculated by multiplying the x/y dimensions of the simulation box and divided by the number of lipid and cholesterol molecules present in one leaflet of the bilayer. The bilayer thickness was calculated as the distance between the phosphate peaks in the electron density profile. For CG (42 nm) systems, area per lipid and bilayer thickness were calculated using Voronoi technique (implemented in APL@Voro)^47^. The two approaches gave comparable results (within the errors) for area per lipid and thickness in absence of bilayer oscillation (see *CG-Reference*(*7* *nm*) values in Table S2).

The order parameters (*S*_*C*_) for carbon were defined as:

1 $${S}_{C}=\frac{1}{2}\langle 3{\cos }^{2}{\theta }_{i}-1\rangle $$

where *θ*_*i*_ is the angle between the molecular axis given *C*_*i*−1_ and *C*_*i*+1_ carbon atoms and the lipid bilayer normal. Eq. 1 may not be the most appropriate to reproduce experimental deuterium order parameters^48^, but it allows to quantify the difference in lipid tail flexibility.

To examine the orientation of cholesterol molecules in the membrane models, we calculated the angle between the cholesterol vector (defined by the carbon atom bonded to the hydroxyl group (OH) and the carbon atom bonded to the acyl tail of the cholesterol) and the bilayer normal (z-axis).

The presence of hydrogen bonds was evaluated using distance-angle criteria: distance between the donor and acceptor and the angle between the donor, hydrogen, and acceptor atoms. The cut-off values were 0.35 nm (for distances) and 135° (for angles).

To identify lipid interdigitation, the mass overlap between upper and lower leaflets was calculated using lipid interdigitation tool in Membplugin^49^.

To quantify the water penetration in the lipid bilayers, we calculated the probability to find a water molecule in the membrane plane at hydrophobic center of the bilayer. A zero-value means that no water molecule was observed in the sub-volume during data production and a non-zero value means that a water has been observed in the volume for a time window. The bin size was chosen 0.31 nm to have 1 water molecule per volume of cube (number density of water is 33.36 nm^−3^ in the bulk water).

The software VMD^50^ was used for graphical representations.

### Mechanical properties

To see the difference on the cohesive properties of membrane models, we calculated area compressibility modulus (*K*_*A*_) from the derivative of surface tension as function of the areal strain,

2 $${K}_{A}={(\frac{\partial \gamma }{\partial {\varepsilon }_{A}})}_{T}=\frac{{K}_{B}T{\langle A\rangle }_{eq}}{{\langle \delta {A}^{2}\rangle }_{eq}}$$

where *γ* is the surface tension and *ε*_*A*_ is the areal strain, defined as ${\varepsilon }_{A}=(\frac{A}{{A}_{0}})-1$ (where A is the average bilayer surface area at specific surface tension and *A*_0_ is the average surface area at zero surface tension). To calculate area compressibility modulus, we used NP_*z*_*γ*T simulations at a tension regime (*γ* = 2, 5 and 10 mN/m) in line with experimental approach^16,51^. We divided NP _*z*_*γ*T production data in 4 parts and calculated the average *γ* and *ε*_*A*_ values. We performed linear regression between the obtained *γ* and *ε*_*A*_ values, the slope of the regression line is *K*_*A*_ and the standard error of the slope is the standard error of *K*_*A*_. The fitting R-squared values are always greater than 0.9. *K*_*A*_ can also be evaluated from the average (〈A〉) and mean square fluctuation (〈*δA*^2^〉) of bilayer surface area at equilibrium (right side of Eq. 2). We checked that the two approaches gave comparable results: *K*_*A*_ for the *CG-Reference* (*7* *nm*) membrane is 457 mN/m with the first approach and 465 mN/m with the second approach. We have observed similar agreement for other membrane models. Further, only the values obtained from the surface tension-areal strain slope are reported.

The bending modulus (*K*_*c*_) was calculated only for *Pure-POPC* membrane models using the following equation^51^:

3 $${K}_{c}=\frac{{K}_{A}{(h-{h}_{0})}^{2}}{24}$$

where *h* is the bilayer thickness and *h*_0_ is equal to 1 nm. Note that assumption *h*_0_ = 1 nm is valid for pure PC bilayers in liquid phase^51^.

## Results and Discussion

### Membrane systems at equilibrium

The membranes have been described as lipid bilayers and simulated at atomistic and CG levels. Different lipid compositions have been considered (see Table 1 for details). We are interested in the effect of SM and GalCer lipids on the membrane structural features. In general, area per lipid shows the following trend *GalCer-rich* ~ *SM-rich* < *Reference* < *POPC/POPE*, while bilayer thickness has an opposite trend *GalCer-rich* > *SM-rich* ~ *Reference* > *POPC/POPE* (Table S2). No major difference in area per lipid is observed for *SM-rich* and *GalCer-rich*, except for CG (42 nm) where a slighly lower value is observed in the presence of GalCer. *SM-rich* and *GalCer-rich* area per lipid is around 0.43–0.48 nm^2^ (Table S2). A values of 0.40 nm^2^ was previously reported^52^ for SM lipid bilayers with 30% cholesterol using MD simulation and an united atom force field. To separate the role of cholesterol, we have compared the cholesterol-containing bilayers with *POPC/POPE* (no cholesterol). On average, the presence of cholesterol decreases the area per lipid of 21% and slightly increases the membrane thickness of around 7% in agreement with the NMR data^53^. The presence of GalCer lipids slightly enhances this effect when it is compared with *Reference* membrane (the area per lipid decreases of around 6% and the membrane thickness increases of 3%), while SM lipids affect only the area values. Both atomistic and coarse-grained models show similar trends, but the coarse grained models show systematically larger values for area per lipid and smaller for thickness than the atomistic simulations as previously reported^33^.

**Figure 2:**
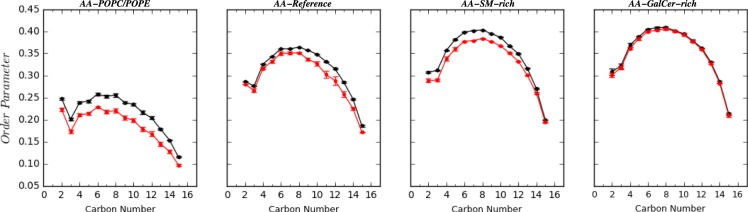
Lipid tail order parameters for sn1 chain as function of carbon number for POPC lipid molecules (for molecular sketch see Fig. S1) in different bilayer models at equilibrium (black line) and 10 mN/m surface tension (red). See Figs S2 and S5 for another chain and other surface tension values.

To study the effect of SM and/or GalCer lipids on the flexibility of carbon tails, we calculated the order parameters for carbon atoms of POPC lipid tails in different bilayers (Figs 2 and S2). The presence of SM or GalCer lipids reduces the dynamics of the lipid tails, but this effect is smaller than the one observed for cholesterol. As previously reported^54^ and observed in our simulations, the presence of cholesterol molecules increases the carbon tail order parameters. Interestingly, the cholesterol angles in *SM-rich* and *GalCer-rich* bilayers have narrower distributions than in *Reference* system, in line with higher order of hydrocarbon chains in these systems (Fig. S3). Experimentally, pure POPC and pure POPE bilayers are in the liquid-disordered phase at 37 °C^55,56^, while the addition of 30% cholesterol promotes a liquid-ordered phases. For mixtures containing sphingomyelin/POPC/CHOL both liquid-disordered and liquid-ordered phases have been observed at 37 °C^57^. Coexistence of liquid-disordered and liquid-ordered phases in ternary lipid systems have also been reported using AA simulations^58^.

To elucidate better the local interactions between the lipid molecules, we have looked at the hydrogen bond propensity in atomistic bilayers. We have observed more hydrogen bonds between the lipids in *GalCer-rich* (202 ± 4) bilayer than in *SM-rich* (154 ± 1) and *Reference* (81 ± 2). GalCer and SM lipids differ in propensity to form hydrogen bonds, in particular GalCer lipids have higher tendency to form hydrogen bonds with POPC lipids and with themselves (GalCer) than SM lipids (Table S3). The sugar hydroxyl groups of GalCer can hydrogen-bond the POPC phosphate group (Fig. 1c) and/or the sugar ring of another GalCer lipid (Fig. 1d). The H-bond network between GalCer-POPC lipids pushes POPC lipids out of the bilayer (as shown by the density maps in Fig. S4) resulting in an increase of thickness (Table S2) and SAS (Fig. S6) for *GalCer-rich* in comparison to *SM-rich*. GalCer sugar rings also have the tendency to pack (Fig. 1d) to each other. On average the center of mass distance between two GalCer sugars is around 0.6 nm allowing sugar ring hydrophobic packing. Such type of interaction between GalCer lipids was previously observed in MD simulations of a mixed bilayer containing GalCer^59^. The lipids are also involved in hydrogen bonds with water. One POPE or GalCar lipid forms on average 8 H-bonds with water molecules while one SM or POPC lipid forms 7 (Table S4). Lipid-water hydrogen bond propensity for a lipid molecule is POPE $\simeq$ GalCer > SM $\simeq$ POPC.

**Figure 3:**
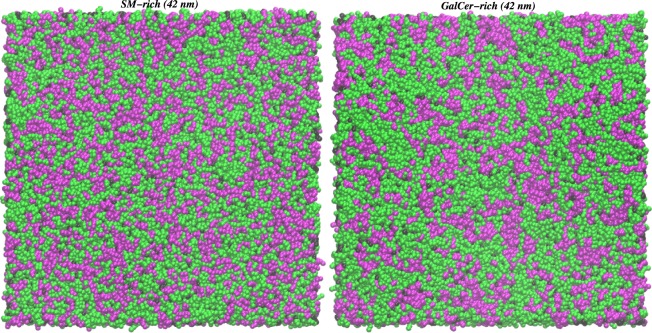
A snapshot at 3 *μ*s of *SM-rich* (left) and *GalCer-rich* (right) CG (42 nm) systems. POPC/POPE molecules in green, CHOL in black, and SM or GalCer in magenta. The water and ion molecules are removed for clarity. See Table S5 for lipid distribution analysis.

At last, we have looked at the lipid distribution in the membrane plane. We have performed the analysis on CG (42 nm) data, since 200 lipid and cholesterol molecules in 7-nm systems may not be enough to identify any pattern between the lipids distribution. By visual inspection, GalCer lipids have slightly higher tendency to be surrounded by other GalCer than SM lipids by SM (Fig. 3). The analysis of the lipid distribution on the bilayer surface (Table S5) shows that on average a GalCer lipid is surrounded by around 5 GalCer lipids, while a SM lipid is surrounded by 4 SM lipids. A similar behaviour has been previously reported for ganglioside lipids (galactosylceramide-like lipids with extra sugars in the headgroup) in CG simulation studies^23,60^. Note that domains at micro scale level can form in the quaternary bilayers and 42-nm system (containing 7200 lipid and cholesterol molecules) is too small to show them.

In summary, both SM and GalCer lipids influence the membrane structure by slightly reducing the area per lipid and increasing the order in the aliphatic tails. GalCer headgroups tight aggregate *via* sugar-sugar interactions and form H-bonds with the POPC headgroups. This results in *GalCer-rich* bilayers being thicker than *SM-rich* and *Reference* bilayers.

### Mechanical properties of membrane models

**Figure 4:**
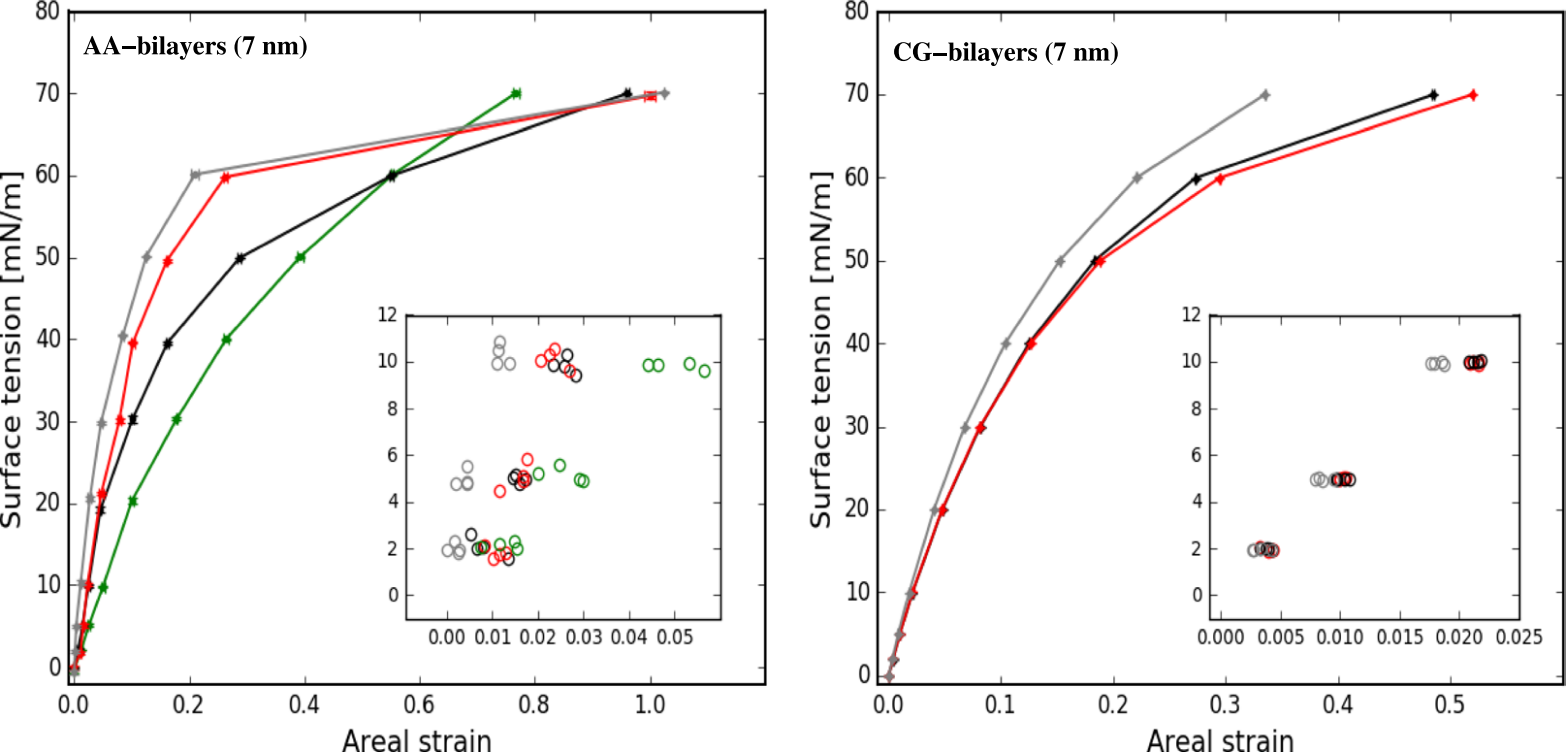
Surface tension as function of the areal strain for bilayer models: *Reference*, *SM-rich*, and *GalCer-rich* are colored in black, red, and gray, respectively. Insets show the values at small areal strain values obtained by dividing the production run in four time-windows. For atomistic model, *Pure-POPC* (in green) is reported for comparison.

To study the response of membranes to the mechanical stress, simulations have been performed at several surface tensions. Figure 4 reports the surface tension as a function of the areal strain for each membrane model. The curves follow similar trend in AA and CG descriptions. In general, the surface tension first increases linearly (see insert in Fig. 4), then it follows logarithmic-like behaviour up to a plateau. The *GalCer-rich* membrane shows the steepest slope of surface tension-areal strain. That means that a membrane containing GalCer is more resistant to change its area in response to surface tension than a GalCer-less membrane at the same concentration of cholesterol.

Area compressibility modulus, *K*_*A*_, has been calculated for each bilayer type and reported in Table 2. The results show a clear trend in *K*_*A*_ values due to the addition of GalCer lipids (*Reference* < *GalCer-rich*). Here, molecular description (AA vs CG) and system size (200 vs 7200 molecules) agree on the qualitative level, but not on the quantitative level. For *SM-rich*, the trends are less clear and the values slightly depend on the molecular description and system size: *K*_*A*_ values are between *Reference* and *GalCer-rich* for AA system, close to *GalCer-rich* in CG (42 nm) system and close to *Reference* for CG (7 nm).

**Table 2:** Area compressibility modulus (*K*_*A*_) and bending modulus (*K*_*c*_) for different membrane models at 310 K.

Membrane Bilayers	AA (7 nm)	CG (7 nm)	CG (42 nm)
***K***_***A***_ **(mN/m)**			
*Pure-POPC*	188 ± 18	297 ± 3	282 ± 2
*POPC/POPE* ^a^	191 ± 16	339 ± 3	306 ± 2
*Reference*	401 ± 48	457 ± 9	360 ± 22
*SM-rich*	532 ± 94	456 ± 8	396 ± 6
*GalCer-rich*	701 ± 94	526 ± 16	384 ± 23
***K***_***c***_ **× 10** ^**−20**^ **(J)**			
*Pure-POPC*	6.7 ± 0.7	10.2 ± 0.1	10.1 ± 0.1

^a^Values calculated using average area at equilibrium (*A*_*eq*_) to define *ε*_*A*_. Note, the use of *A*_*eq*_ in place of *A*_0_ affects *K*_*A*_ values within the error in one-to-three component bilayers.

For *Reference* (*7* *nm*) bilayer, the *K*_*A*_ values for AA and CG simulations are 401 and 457 mN/m, respectively, showing an agreement between the two molecular descriptions. But as soon as the lipid composition becomes more complex, the *K*_*A*_ values start to diverge and the atomistic values are systematically larger than the corresponding CG ones. Concerning the system dimensions, the *K*_*A*_ values are systematically smaller for large systems: *Reference* (*42* *nm*) is 360 mN/m, around 90 mN/m less than the corresponding 7-nm system, and the same trend is also observed for *GalCer-rich* systems. Further, larger area fluctuations (〈*δA*^2^〉) are observed for the 42-nm than for the 7-nm membranes, reflecting a decrease in area compressibility modulus (see Eq. 2). Effect of bilayer size on *K*_*A*_ has been previously reported by Waheed *et al*.^61^ in a simulation study of phosphatidylcholine lipid bilayers containing 256, 1024, and 2304 lipid molecules. Note that we can not exclude that 42-nm system (containing 7200 lipid and cholesterol molecules) may be too small to allow the formation of naturally occurring domains, and this may have some effects on the *K*_*A*_ values.

### Mechanical properties - comparison with experiments

As far as we know, no experimental data (area compressibility and bending modulus) are available for *SM-rich* and *GalCer-rich* bilayers to directly compare our results, but experimental data are available for *Pure-POPC*^10,51,62^ and on the effect of cholosterol^16,63^ on *K*_*A*_ values (Table S6). Thus, we have simulated *Pure-POPC* and *POPC/POPE* systems at atomistic and CG levels and calculated the corresponding *K*_*A*_ and *K*_*c*_ (Table 2), to allow a comparison with the available experiment data.

The calculated area compressibility modulus *Pure-POPC* is 188 mN/m at AA level and 282–297 mN/m at CG level, and the bending modulus is 7 and 10 ×10^−20^ J, respectively for AA and CG models (Table 2). Experimentally, area compressibility modulus for POPC bilayers is reported in a range between 208 and 237 mN/m at 21 °C using micropipette aspiration^51^ and between 180 and 330 mN/m at 25 °C using infrared measurements^62^, while the bending modulus varies between 9 and 10 × 10^−20^ J^10,51^. Both AA and CG *K*_*A*_ values are in the experimental range, while bending modulus is better reproduced by MARTINI force field than by the CHARMM36 force field.

We have also looked at the effect of cholesterol on the area compressibility modulus. Simulations show that having 30% of cholesterol molecules in the membrane composition increases *K*_*A*_ of around 110% (191 mN/m for *POPC/POPE* and 401 mN/m for *Reference*) at atomistic level and 15–35% for CG models (Table 2). Micropipette aspiration experiments conducted on lipid vesicles showed that the cholesterol concentration increase the membrane stiffness (Table S6)^16,63^. For example, Needham *et al*.^16^ observed an increases (75%) of the area compressibility modulus when 38% cholesterol is added to phosphocholine vesicles (Table S6). Experiments and simulations agree that cholesterol molecules cause an increase in membrane compressibility modulus, accounting for difference in temperature measurements, compositions, and techniques.

When SM lipids are added, *K*_*A*_ increases 33% and 10% for AA and CG (42 nm) descriptions, respectively, while almost no effect is observed for CG (7 nm). An increase of *K*_*A*_ in presence of SM lipids was previously observed in micropipette experiments and in simulation studies^16,64^. For example, micropipette experiments reported higher *K*_*A*_ value for the bovine sphingomyelin vesicle with 50% cholesterol (1718 mN/m) than for PC vesicle with the same amount of cholesterol (781 mN/m)^16^. Simulation data on pure SM bilayer showed larger *K*_*A*_ value (310 mN/m) than for pure POPC and POPE lipid bilayers (260 mN/m and 280 mN/m)^64^. Berta Gumí-Audenis and coworkers^18^ reported larger breakthrough force by increasing GalCer and/or SM concentrations in membranes containing cholesterol using AFM-based force spectroscopy. All together the experimental evidences support that adding SM or GalCer to a bilayer increases bilayers stiffness in line with our AA and CG (42 nm) simulations.

Experiments and simulations agree that lipid compositions have an effect on bilayer compressibility. Having cholesterol in the bilayer increases the stiffness of the membrane and adding GalCer lipids to the bilayer enhances the resistance to extension. Unluckily, the experimental variation in the area compressibility modulus hinder quantitative assessment of simulation models. The comparison, accounting for the limited number of experimental data, indicates that a better agreement between experiment and simulation is achieved either using at least 42-nm bilayers (7200 molecule bilayers) and CG description or small bilayers (200 molecules) and a detailed atomistic description.

### Membrane under tension: water penetration and bilayer structure

To describe how mechanical stress alters *GalCer-rich* and *SM-rich* bilayers structure, we have grouped the structures based on the relation between surface tension and areal strain: (1) linear relation, surface tensions less than 10 mN/m, (2) logarithmic-like relation, intermediate values, and (3) constant surface tension (plateau), high values (Fig. 4).

At small surface tension values (<10 mN/m), an increase of surface tension corresponds to a decrease of order in the aliphatic tails (Fig. 2) and an increase to the bilayer surface area. The effect on the aliphatic tails is larger for cholesterol-less systems than for systems containing 30% cholesterol. Almost no difference in the POPC tail order is observed for *GalCer-rich* between 0 and 10 mN/m surface tensions. At these surface tensions, SAS for *GalCer-rich* bilayer is constant, while we observe a slightly increase for the *SM-rich* bilayer when the surface tension increases (Fig. S6). No change in the bilayer density profile and no water penetration is observed in this group.

**Figure 5:**
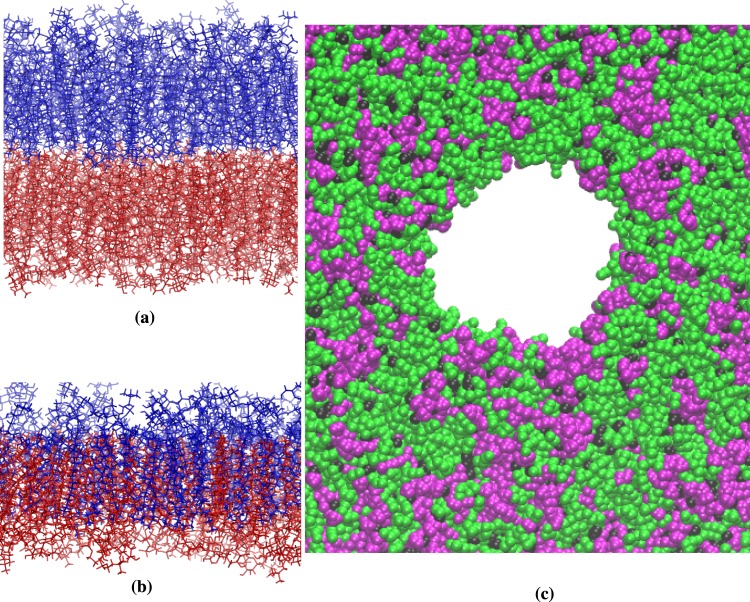
Representative snapshots for *GalCer-rich* membrane. (**a**) Equilibrium and (**b**) interdigitation (at 70 mN/m surface tension) states at 0.6 *μ*s of atomistic simulation. The lipid molecules in upper and lower leaflets are shown in blue and red colors, respectively. (**c**) Close-up of a pore from *CG-GalCer-rich* (*42* *nm*) at 0.15 *μ*s of simulation. POPC/POPE molecules are shown as green, CHOL as black, and GalCer as magenta with VDW representations. The water and ion molecules are removed for clarity.

At surface tension >10 mN/m, the relation between the surface tension and area becomes logarithmic-like. The lipid tails are more disordered and the bilayer density profiles change (Figs S5 and S7). Water profile shifts toward the hydrophobic center of the bilayer while the maximum of lipid profile decreases and moves toward the center of bilayer, causing a decrease in membrane thickness. At the plateau values, the order of end of the aliphatic tails increases (Fig. S5), the overlap between the leaflets increases (Fig. S7) and a fully interdigitated state is observed for the bilayers (Fig. 5b). Membrane interdigitation is usually observed in presence of amphiphilic molecules and/or due to change in hydrostatic pressure or in the pH of the membrane^65–67^. We speculate that the plateau values are a sort of transition point for the membrane structural and mechanical properties. The transition point is around 70 mN/m for *AA-SM-rich* and *AA-GalCer-rich* membranes, while bilayers interdigitation is not observed for the corresponding CG models up to 70 mN/m. We have performed an extra simulation for the *CG-GalCer-rich* (*7* *nm*) system at higher surface tension than 70 mN/m and observed interdigitation at surface tension 80 mN/m (see inserts in Fig. S7).

**Figure 6:**
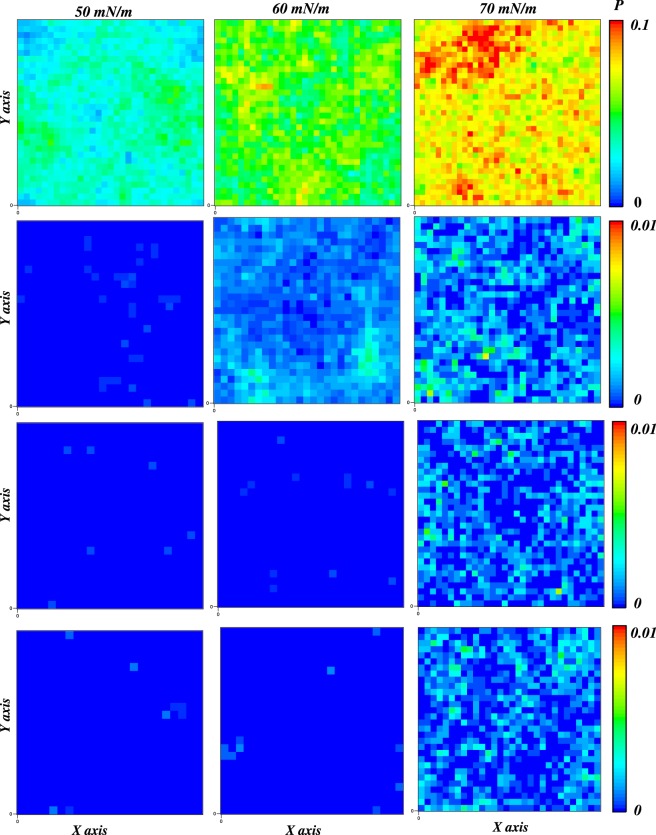
Probability map of finding a water molecule in the hydrophobic center of the bilayer at different surface tensions (from 50 to 70 mN/m) for *AA-Pure-POPC* (first row), *AA-Reference* (second row), *AA-SM-rich* (third row), and *AA-GalCer-rich* (fourth row) bilayers. Note a different scale is used for *AA-Pure-POPC* data.

To evaluate how mechanical stress influences *SM-rich* and *GalCer-rich* water permeability, we have calculated the probability to find a water molecule in the hydrophobic core of the bilayers. Up to a surface tension of 60 mN/m, less than 2% of the bilayer experiences a water molecule in the hydrophobic core (see Fig. 6 for the atomistic models) for a period of time. At a surface tension of 70 mN/m, the value increases to 57% (*SM-rich*) and 65% (*GalCer-rich*). Interestingly, at this surface tension, we start to see bilayer interdigitation for atomistic simulation. In summary, the water penetration propensity at the same surface tension is: *GalCer-rich* = *SM-rich* < *Reference* membrane. The results agree with a previous membrane simulation results^52^ that reported an increase for free energy barrier of water permeation in SM/CHOL bilayer compared to the PC/CHOL bilayer. The presence of SM and/or GalCer lipids enhances the effect observed previously for cholesterol^52^: a decrease in the water permeability (for comparison *AA-Pure-POPC* data are reported in Fig. 6).

As the surface tension exceeds a value of 70 mN/m, the number of water molecules inside the bilayer increases. This poses the question at which surface tension a pore will form in the bilayer. To monitor pore formation, we have simulated CG membranes (42 nm) at different constant areal strains (NP_*z*_AT ensemble), starting from 0.5. At 0.8 areal strain a pore is formed. The average surface tension before the rupture is similar for *CG-Reference* (*42* *nm*) and *CG-SM-rich* (*42* *nm*) (76.4 mN/m and 75.6 mN/m, respectively) but slightly higher for *CG-GalCer-rich* (*42* *nm*) (80.0 mN/m). From visual inspection of the pore, we observe that SM and GalCer lipids are located at the bilayer-water-pore interface (Figs 5 and S8).

In summary, we observe the similar pattern in the structural change at the increase of surface tension for all bilayers, independently of the lipid content. The lipid composition modulates at which surface tension the transitions take place. In general, the presence of GalCer and/or SM increase bilayer resistance to water penetration.

## Conclusion

We have investigated the influence of lipid compositions on membrane structural and mechanical properties using molecular dynamics simulations. Lipid bilayers with different compositions have been modelled using both atomistic and CG descriptions. The composition of the bilayers were chosen such that they reflect the difference between myelin sheath and other types of membranes. In particular a GalCer/POPC/POPE/CHOL bilayer was used to represent the myelin sheath and compared with SM/POPC/POPE/CHOL or POPC/POPE/CHOL bilayers.

Atomistic and CG simulations agree on how sphingomyelin and galactosylceramide lipids influence the membrane structural properties. In general, both sphingomyelin and galactosylceramide lipids cause a decrease in the area per lipid and an increase in the order parameter of the lipid tails, enhancing effects already observed for cholesterol molecules.

Constant surface tension simulations have been used to mimic the effect of mechanical stress on the membrane. For all investigated membranes, the surface tension first increases linearly with the areal strain, then in a logarithmic-like way, and finally, reaches a plateau. The derived area compressibility modulus, *K*_*A*_, depends on the lipid content. In particular, adding galactosylceramide lipids increase the bilayer resistance to extension.

Galactosylceramide lipids tend to pack *via* sugar-sugar interaction and to form hydrogen bonds with POPCs. This results in POPC being pushed slightly out of the bilayer surface and *GalCer-rich* bilayers being thicker and more cohesive than other bilayers. Moreover, galactosylceramide lipids make the membrane more resistant to water penetration than phospholipids. Membrane interdigitation is observed at a surface tension >70 mN/m and pore formation at areal strain around 0.8.

In conclusion, the results show a clear role of galactosylceramide lipids on the structural and mechanical properties of the bilayers. Since galactosylceramide lipids are peculiar for myelin sheath, the results contribute to have a better molecular insight in role of lipid composition in the axonal membrane and on the possible relation between lipid content and protective function in myelin sheath.

## Supplementary information

Supplementary Information
