# Supplementary material for: Role of lipid composition on the structural and mechanical features of axonal membranes: a molecular simulation study
Source: Sci Rep. 2019 May 29;9:8000. doi: 10.1038/s41598-019-44318-9 (PMC6541598; doi:10.1038/s41598-019-44318-9)
Supplement: Supplementary file 1 — Supplementary Information [file 41598_2019_44318_MOESM1_ESM.pdf]

Role of lipid composition on the structural and mechanical features of axonal membranes: a molecular simulation study

Marzieh Saeedimasine[1], Annaclaudia Montanino [2], Svein Kleiven[2], and Alessandra Villa[1,*]

[1] *Department of Biosciences and Nutrition, Karolinska Institutet, Huddinge, Sweden*

[2] *Division of Neuronic Engineering, KTH-Royal Institute of Technology, Huddinge, Sweden*

[*]*Corresponding authors:* alessandra.villa@ki.se

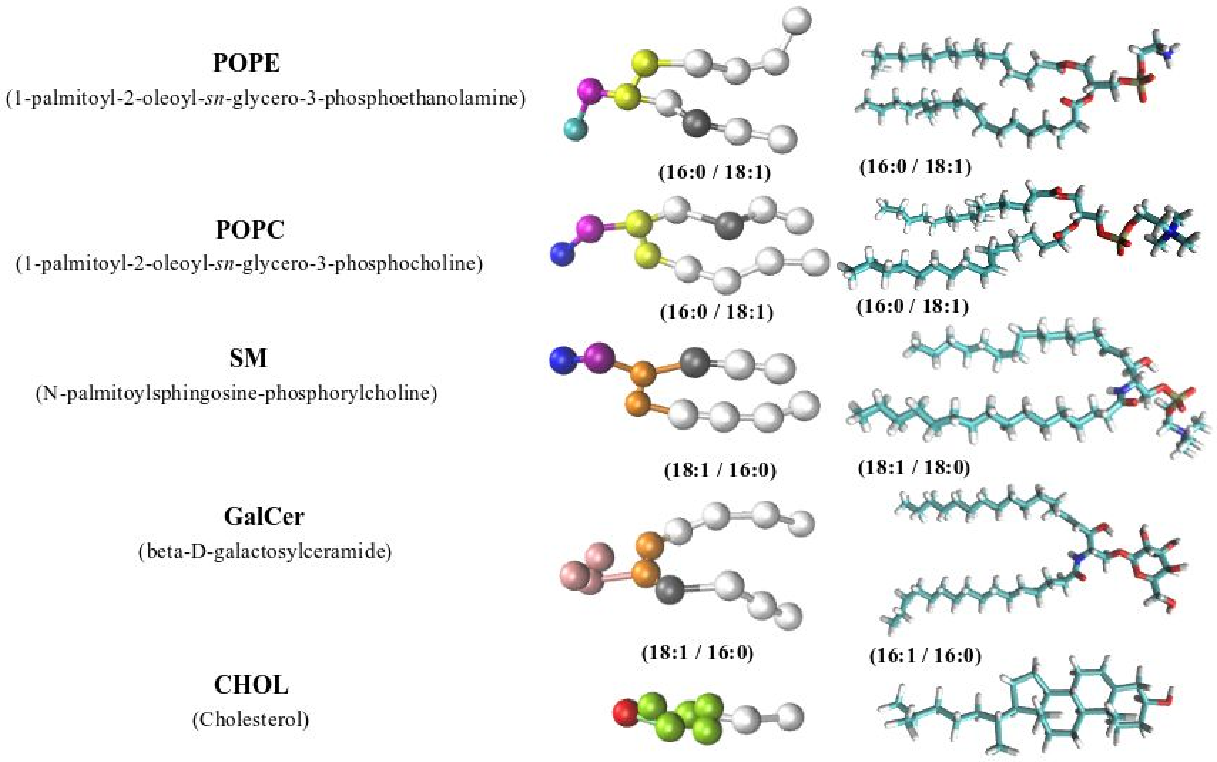

Figure S1: Molecules abbreviations and names, atomistic and coarse grained description chemical structures together with lipid tail (sn1:sn2) definition. AA structures are shown in **Licorice** representation and colored as follow, carbon are colored in cyan, oxygen is red, hydrogen is white, nitrogen is blue, and phosphorus is brown. CG structures are shown in **CPK** representation as follow, apolar is white or gray, nonpolar is yellow, polar is orange, pink or green, and charged is colored by blue, magenta, cyan, or red.

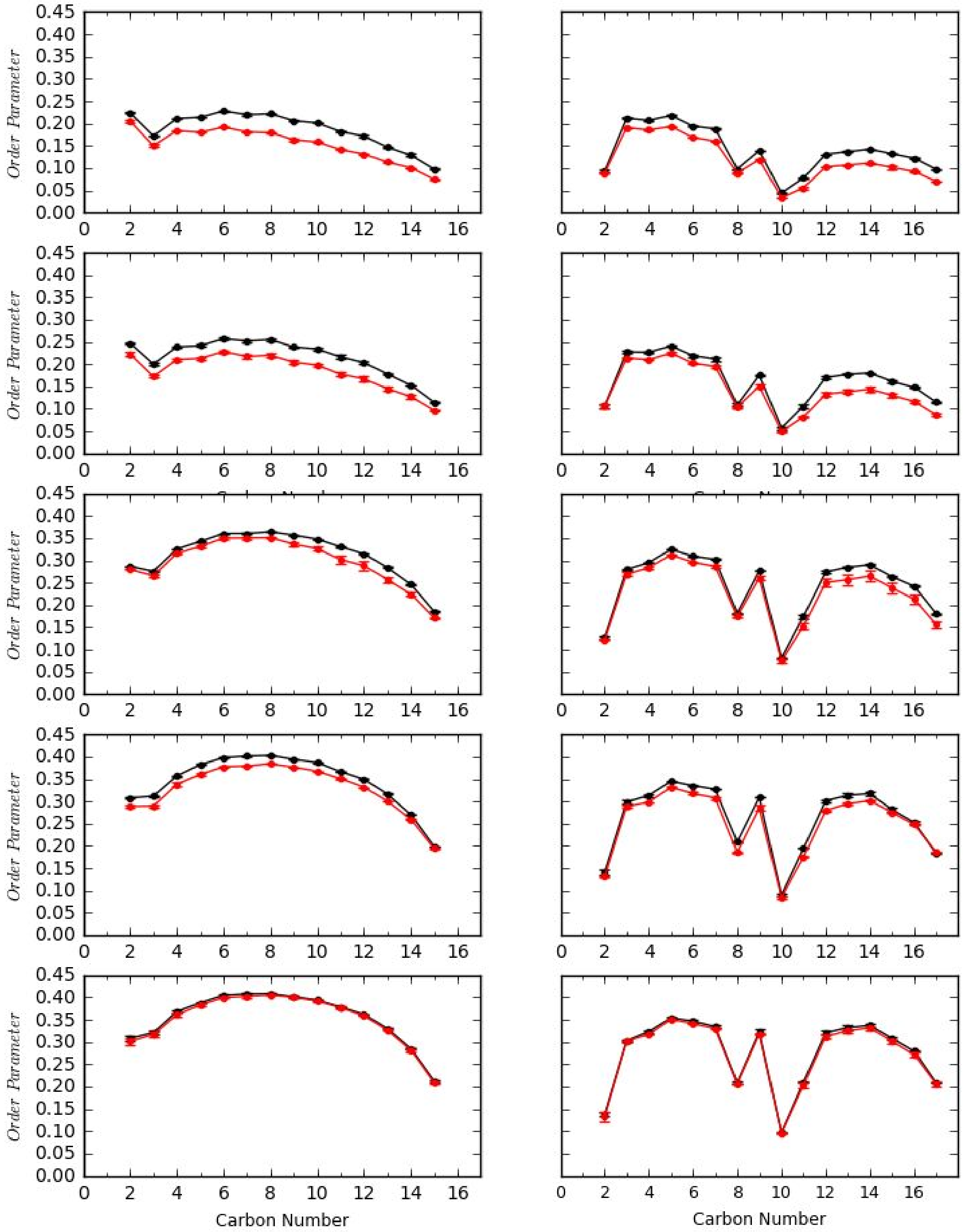

Figure S2: Lipid tail order parameters for sn1 (left side) and sn2 (right side) chain as function of carbon number for POPC lipid molecules in different membrane models, ***AA-Pure-POPC*** (first row), ***AA-POPC/POPE*** (second row), ***AA-Reference*** (third row), ***AA-SM-rich*** (fourth row), and ***AA-GalCer-rich*** (fifth row) at equilibrium (black line) and 10 mN/m surface tension (red). The errors were obtained by dividing the data production into four parts and calculating the standard error between them.

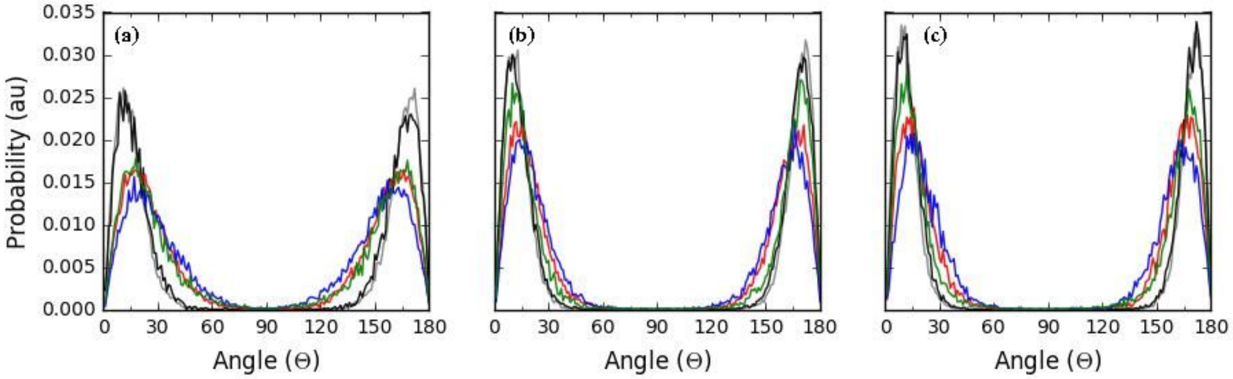

Figure S3: Angular distribution of cholesterol vector relative to the bilayer normal (z-axis) in ***AA-Reference*** **(a)**, ***AA-SM-rich*** **(b)**, and ***AA-GalCer-rich*** **(c)** membranes for equilibrium (gray), 10 (black), 50 (red), 60 (blue), and 70 (green) surface tension values (mN/m).

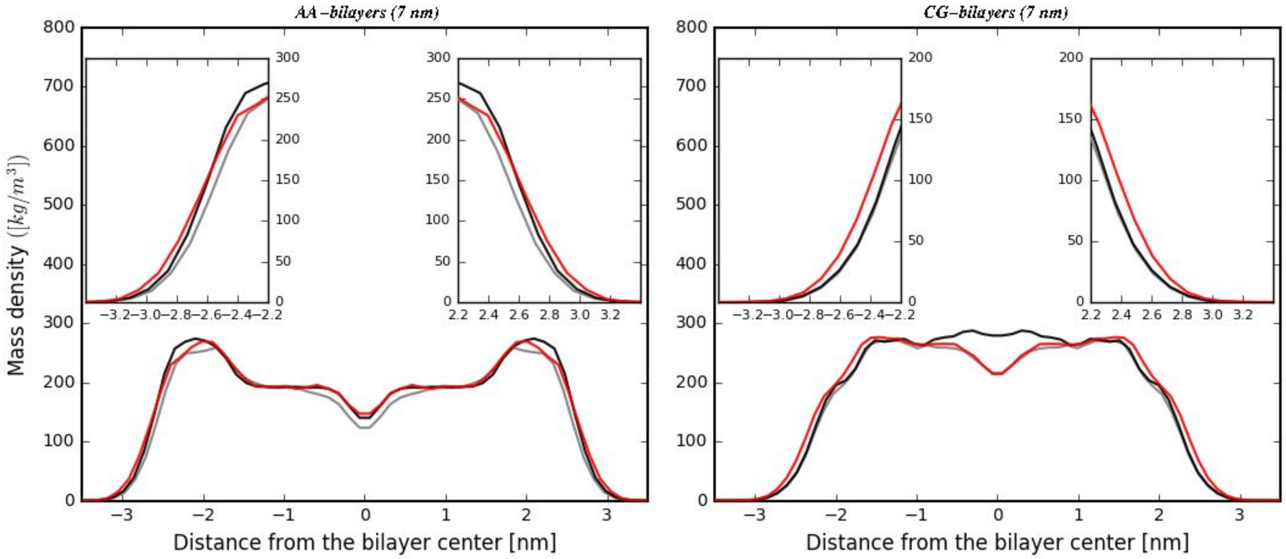

Figure S4: Mass density distribution of POPC lipids for ***Reference*** (gray) **,** ***SM-rich*** (black) and ***GalCer-rich*** (red) membrane models. In insets, mass density distributions between 2.2 to 3.2 nm from the bilayer center.

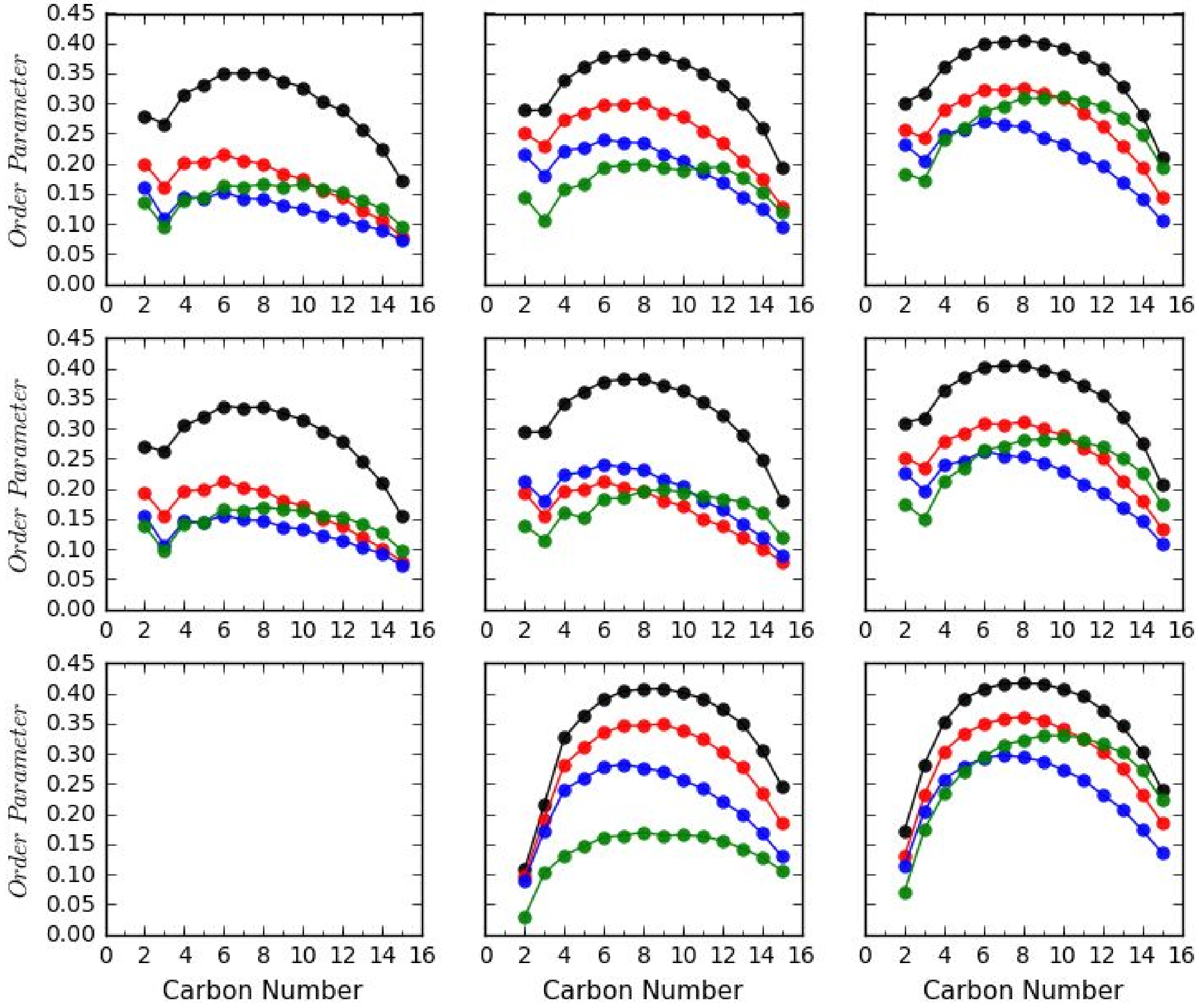

Figure S5: Lipid tail order parameter of sn1 chain as function of carbon number for POPC (first row), POPE (second row), and PSM or GalCer (third row) lipid molecules in ***AA-Reference*** (left), ***AA-SM-rich*** (middle), and ***AA-GalCer-rich*** (right) side of the figure. Different surface tension values are colored as follow: 10 (black), 50 (red), 60 (blue), and 70 (mN/m) (green).

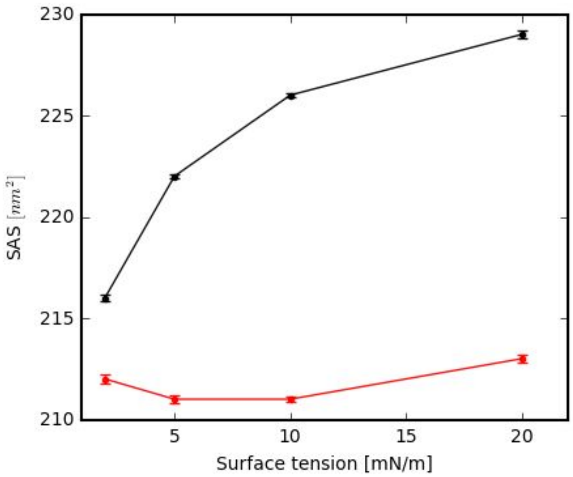

Figure S6: Solvent accessible surface area as function of the surface tension (from 2 to 20 mN/m) for **AA-*SM-rich*** (black) and **AA-*GalCer-rich*** (red) membranes. The errors were obtained by dividing the trajectories of data production into four parts and calculating the standard error between them.

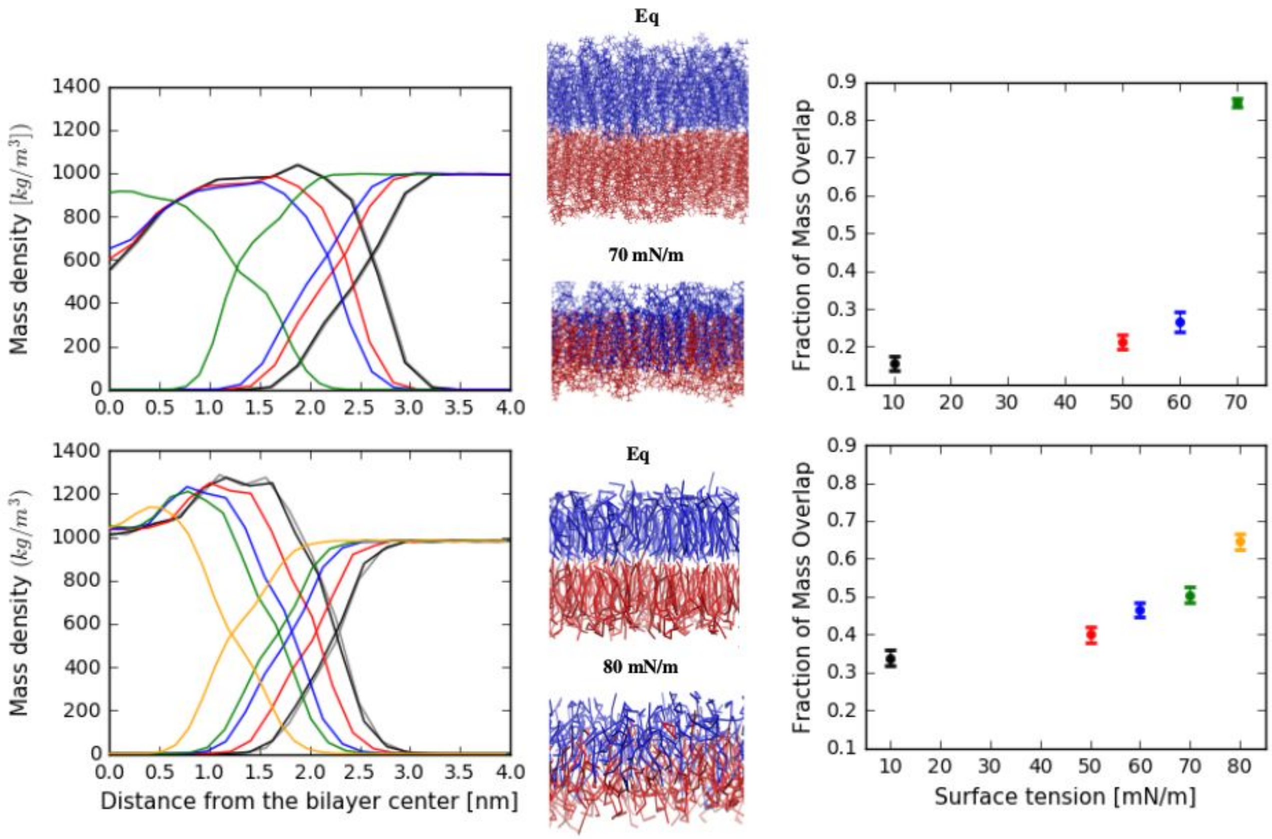

Figure S7: Mass density distribution of water and lipid molecules (left side) and leaflets mass overlap (right side) for ***AA-GalCer-rich*** (7 nm) (up) and ***CG-GalCer-rich*** (7 nm) (down) bilayers. Equilibrium (gray), 10 (black), 50 (red), 60 (blue), 70 (green), and 80 (orange) surface tension values (mN/m). z= 0 corresponds to the bilayer center. Fraction of two leaflets mass overlap is reported as function of surface tension. To calculate leaflet overlap, the tool in Membplugin (Guixà-González, 2014, https://doi.org/10.1093/bioinformatics/btu037) was used.

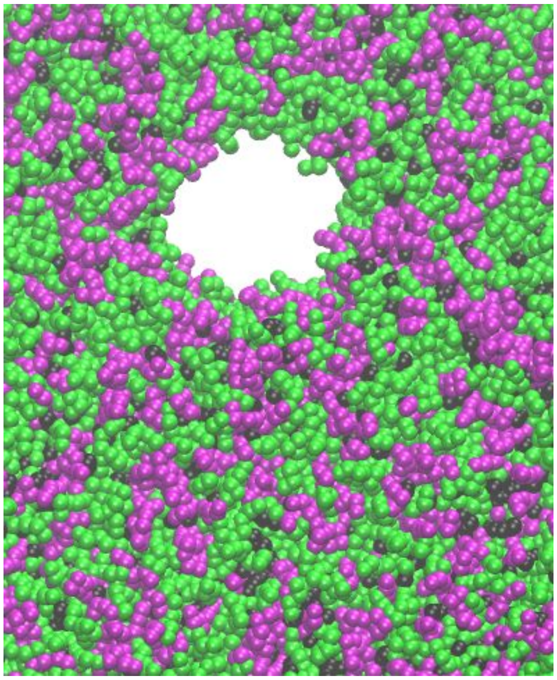

Figure S8: Close-up of a pore in ***CG-SM-rich*** (42 nm) simulation at 0.016 μs. POPC and POPE molecules are shown as green, CHOL as black, and SM as magenta with VDW representations. The water and ion molecules are removed for clarity.

Table S1: Molecular simulation details: For each simulation values of surface tension (mN/m), simulation time (µs) and molecular model and x/y-dimension are reported. The label AA and CG are for atomistic and coarse grained model, respectively.

**Models/ Surface tension (mN/m)**	**Equilibrium**	**0**	**2**	**5**	**10**	**20**	**30**	**40**	**50**	**60**	**70**	**80**
**AA- *Pure-POPC* (7 nm)**	0.6	0.4	0.6	0.6	0.6	0.6	0.6	0.6	0.6	0.6	0.6	-
**AA- *POPC/POPE (7 nm)***	0.4	-	0.4	0.4	0.4	-	-	-	-	-	-	-
**AA-*Reference* (7 nm)**	0.6	0.4	0.6	0.6	0.6	0.6	0.6	0.6	0.8	0.8	0.8	-
**AA-*SM-rich* (7 nm)**	0.6	0.4	0.6	0.6	0.6	0.6	0.6	0.6	0.8	0.8	0.8	-
**AA-*GalCer-rich* (7 nm)**	0.6	0.4	0.6	0.6	0.6	0.6	0.6	0.6	0.8	0.8	0.8	-
**CG- *Pure-POPC* (7 nm)**	30	30	30	30	30	-	-	-	-	-	-	-
**CG-*POPC-POPE* (7 nm)**	30	-	30	30	30	-	-	-	-	-	-	-
**CG-*Reference* (7 nm)**	50	50	50	50	50	50	50	50	50	50	50	-
**CG-*SM-rich* (7 nm)**	50	50	50	50	50	50	50	50	50	50	50	-
**CG-*GalCer-rich* (7 nm)**	50	50	50	50	50	50	50	50	50	50	50	50
**CG- *Pure-POPC* (42 nm)**	3	3	3	3	3	-	-	-	-	-	-	-
**CG-*POPC-POPE* (7 nm)**	3	-	3	3	3	-	-	-	-	-	-	-
**CG-*Reference* (42 nm)**	3	3	3	3	3	3	3	3	3	3	3	-
**CG-*SM-rich*(42 nm)**	3	3	3	3	3	3	3	3	3	3	3	-
**CG-*GalCer-rich* (42 nm)**	3	3	3	3	3	3	3	3	3	3	3	-

Table S2: Area per lipid and thickness of studied membrane models. The values for ***CG (42 nm)*** systems are calculated using voronoi algorithm implemented in Voronoi-based membrane analysis tool (APL@Voro). Reported errors are obtained by dividing the data production into four parts and calculating the standard error between them.

**Models**	**Area per lipid (nm^2)**	**Thickness (nm)**
**AA-*Pure-POPC* (7 nm)**	0.633 ± 0.003	3.93 ± 0.01
**AA-*POPC/POPE* (7 nm)**	0.594 ± 0.003	4.13± 0.01
**AA-*Reference* (7 nm)**	0.458 ± 0.003	4.55 ± 0.01
**AA-*SM-rich* (7 nm)**	0.432 ± 0.001	4.68 ± 0.01
**AA-*GalCer-rich* (7 nm)**	0.427 ± 0.002	4.73 ± 0.02
**CG-*Pure-POPC* (7 nm)**	0.657 ± 0.001	3.87 ± 0.03
**CG-*POPC/POPE* (7 nm)**	0.634 ± 0.001	3.93 ± 0.04
**CG-*Reference* (7 nm) [(a)]**	0.505 ± 0.003	4.14 ± 0.01
**CG-*SM-rich* (7 nm)**	0.486 ± 0.003	4.12 ± 0.01
**CG-*GalCer-rich* (7 nm)**	0.481 ± 0.001	4.23 ± 0.02
**CG-*Pure-POPC* (42 nm)**	0.653 ± 0.001	3.93± 0.01
**CG-*POPC/POPE* (42 nm)**	0.630± 0.001	4.02± 0.01
**CG-*Reference* (42 nm)**	0.496 ± 0.001	4.21 ± 0.01
**CG-*SM-rich* (42 nm)**	0.482 ± 0.001	4.17 ± 0.01
**CG-*GalCer-rich* (42 nm)**	0.469 ± 0.001	4.29 ± 0.01

[(a)] For comparison values calculated using Voronoi algorithm are 0.503 ± 0.001 and 4.16 ± 0.01 for area per lipid and thickness, respectively.

Table S3: The average number of lipid-lipid hydrogen bond. Larger differences between ***SM-rich*** and ***GalCer-rich*** are highlighted in yellow. Reported errors are obtained by dividing the data production into four parts and calculating the standard error between them.

	POPC	POPE	CHOL	SM/GalCer
AA-SM-rich				
POPC	0	7.3 ± 0.2	6.1 ± 0.7	7.9 ± 0.7
POPE		15.6 ± 0.8	8.0 ± 0.3	26.3 ± 1.4
CHOL			0	17.9 ± 0.9
SM				64.6 ± 0.7
AA-GalCer-rich				
POPC	0	7.6 ± 0.4	4.9 ± 0.5	32.1 ± 0.8
POPE		16.0 ± 1.0	6.7 ± 0.5	31.0 ± 1.7
CHOL			0	21.8 ± 0.7
GalCer				82.1 ± 1.2

Table S4: The average number of lipid-water hydrogen bond per lipid molecule. Reported errors are obtained by dividing the data production into four parts and calculating the standard error between them.

X-water	POPC	POPE	CHOL	SM	GalCer
Bilayer type					
AA-Pure-POPC	7.24 ± 0.03	-	-	-	-
AA-Reference	6.89 ± 0.01	8.22 ± 0.02	1.60 ± 0.01	-	-
AA-SM-rich	6.68 ± 0.02	7.72 ± 0.07	1.43 ± 0.01	6.82 ± 0.02	-
AA-Gal-rich	6.36 ± 0.04	8.0 ± 0.06	1.48 ± 0.02	-	7.79 ± 0.06

Table S5: Number of neighboring lipids within 1.5 nm for the last 1 μs of CG (42 nm) membrane models. It was calculated by considering the first tail bead after the headgroup of lipid or polar group of cholesterol molecules. Reported errors are obtained by dividing the data production into four parts and calculating the standard error between them.

	POPC	POPE	CHOL	SM/GalCer
SM-rich				
**POPC**	2.51 ± 0.01	2.56 ± 0.01	4.11 ± 0.01	3.80 ± 0.01
**POPE**	2.56 ± 0.01	2.61 ± 0.01	4.22 ± 0.01	3.83 ±0.01
**CHOL**	2.70 ± 0.05	2.80 ± 0.06	3.75 ± 0.16	4.04 ± 0.07
**SM**	2.53 ± 0.01	2.55 ± 0.01	4.11 ± 0.01	3.80 ± 0.01
GalCer-rich				
**POPC**	2.65 ± 0.01	2.69 ± 0.02	3.98 ± 0.01	3.39 ±0.02
**POPE**	2.69 ±0.02	2.69 ±0.02	4.12 ±0.01	3.60 ± 0.02
**CHOL**	2.65 ±0.01	2.75 ±0.01	3.61 ±0.01	4.46 ±0.01
**GalCer**	2.26 ±0.02	2.40 ±0.02	4.46 ±0.01	4.82 ±0.03

Table S6: Collection of experimental data on area compressibility modulus available for phospholipid bilayers.

Lipid Bilayers	K_A (mN/m)	Technique/ Comment
**PC Bilayer**		
diC18:1[(1)]	208-237	Micropipette aspiration/varied between cis and trans unsaturated bonds at 9th carbon. at 21℃
POPC [(2)] (16:0/18:1)	180-330	Infrared measurements/ values refer to change of lateral compression pressure from 0 to 25 mN/m at 25℃
DMPC [(3)] (14:0/14:0)	144.9	Micropipette aspiration/ at 29℃
DPPC [(4)] (16:0/16:0)	250	Estimated value/at 50℃
SOPC [(5, 3)] (18:0/18:1)	190	Micropipette aspiration/ at 18℃
DOPC [(6)] (18:1/18:1)	300	Micropipette aspiration/at 24℃
**PC Bilayer with Cholesterol**		
DMPC+33% CHOL[(3)]	646.8	Micropipette aspiration/ at 15℃
DMPC+40% CHOL[(3)]	600	Micropipette aspiration/ at 35℃
SOPC+38% CHOL[(5)]	333	Micropipette aspiration/ at 15℃

(1) Rawicz, 2000, https://doi.org/10.1016/S0006-3495(00)76295-3

(2) Binder, 2001, https://doi.org/10.1021/jp010118h

(3) Evans,1987, https://doi.org/10.1021/j100300a003

(4) Nagle, 2000, https://doi.org/10.1016/S0304-4157(00)00016-2

(5) Needham, 1990, https://doi.org/10.1016/S0006-3495(90)82444-9

(6) Evans, 2013, https://doi.org/10.1039/C2FD20127E
